# Genes, Genomes, and the Road to Diversity: How Regulatory Networks Evolve

**DOI:** 10.1371/journal.pbio.0020441

**Published:** 2004-11-09

Evolutionary biologists have long been interested in understanding the molecular basis for the great diversity in size, shape, and behavior seen in life on earth. Recent attention has focused on the role that gene expression changes play in organismal evolution. Tracing the evolution of gene regulation, however, has proved difficult. This is in large part due to the difficulty in identifying and comparing the regulatory elements that control gene expression in different species.

Gene expression depends on *cis*-regulatory elements, short sequence motifs embedded in the DNA that flank a gene's coding region. Regulatory proteins bind to specific *cis*-regulatory sequences, and command the activation or repression of the corresponding gene. The challenge in studying the evolution of *cis*-regulatory elements lies in identifying those elements in multiple species. Unlike protein sequences, which are typically a few hundred amino acids long and relatively straightforward to identify in related organisms, *cis*-regulatory elements are often short and can have variations in sequence. This makes it very difficult to distinguish the regulatory elements from the nonfunctional DNA that surrounds them. It is even harder to identify corresponding regulatory elements across species. As the evolutionary distance between species increases, so, too, does the difficulty in identifying corresponding cis-elements in those species.

In this issue of *PLoS Biology*, Audrey Gasch and her colleagues describe a comparative genomics approach that allows them to identify potential *cis*-regulatory elements in thousands of genes across 14 ascomycete fungi whose diversity represents the effects of several hundred million years of evolution. Ascomycetes are a large class of fungi with extremely diverse morphologies, reproductive strategies, and habitats. A divergence dating back 500 million to 1 billion years ago gave rise to three groups: Archaeascomycetes, Euascomycetes, and Hemiascomycetes. The genome of the brewer's yeast, *Saccharomyces cerevisiae*, a hemiascomycete, was completely sequenced in 1995, and that of fission yeast, *Schizosaccharomyces pombe*, an archaeascomycete, in 2002. Since that time, complete genome sequences have been released for more than nine additional hemiascomycetes and three euascomycetes. This gives the authors an opportunity to compare regulatory systems among progressively more distantly related species, on a genomic scale.[Fig pbio-0020441-g001]

**Figure pbio-0020441-g001:**
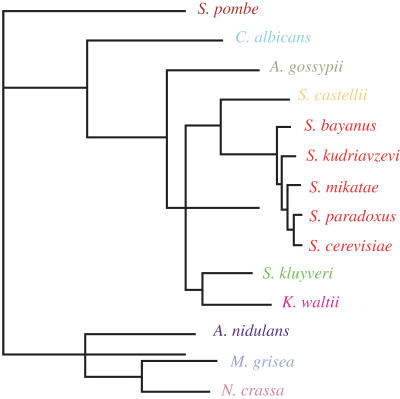
Phylogeny of fungi used to study evolution of gene regulation

Genome-wide expression studies in the yeast *S. cerevisiae* have revealed groups of genes whose expression levels vary simultaneously under varying experimental conditions. Such co-regulated genes, the authors reasoned, must harbor common regulatory elements that coordinate their response to experimental triggers. Gasch and colleagues looked for such *cis*-elements and found 35 groups of co-regulated *S. cerevisiae* genes with at least one shared *cis*-element. The authors then argued that co-regulation may reflect selection pressures that also apply to other ascomycetes, and so they identified the equivalent of the 35 co-regulated gene groups in each of the 13 other species. They then looked for shared *cis*-elements within each group and in each species independently, and compared the regulatory systems across the species.

The results of this study show that the majority of *cis*-elements first identified in yeast are retained in the equivalent gene groups in other species, in a manner that reflects the species' evolutionary distance from yeast. One *cis*-element, in a group of co-regulated genes that control the cell cycle, is found all the way from budding yeast to fission yeast, suggesting a selection pressure on the co-regulation of these genes that has withstood greater than 500 million to 1 billion years of evolution.

In contrast, there were other examples in which the same gene groups contained different putative *cis*-elements in each species, suggesting that the regulation of those genes has evolved. In the case of *cis*-elements found in genes controlling protein degradation, a related element was identified in all of the hemiascomycetes, whereas the euascomycetes appear to have adopted a novel *cis*-element for this gene group. Interestingly, the hemiascomycete element displays a sequence variation in *Candida albicans* that is not found in *S. cerevisiae*. The two species diverged 200 million years ago. Gasch and colleagues showed that the protein that binds to the hemiascomycete element has evolved to have slightly different DNA interactions in the two species, allowing the *C. albicans* protein to bind the novel sequence found only in the *C. albicans* genes. This provides evidence for co-evolution between a transcription factor and its target *cis*-element. Overall, this analysis has uncovered striking cases of conservation and innovation of gene regulatory systems, and therefore provides important insight into the evolutionary forces that have shaped the evolution of gene regulation.

